# P-132. Endocarditis in Northeastern Argentina: 10 Years of Hospital-Based Epidemiological Data

**DOI:** 10.1093/ofid/ofaf695.359

**Published:** 2026-01-11

**Authors:** Lucila Maria Compañy Kec, Gustavo-Adolfo Méndez, Carla Niveyro, Pedro A Villalba apestegui, Victoria Isabel martin, Cynthia Tomasino, Ricardo de Jesus Solari Maidana, Claudia Viviana Villalba

**Affiliations:** Infectious Diseases Department, Hospital Escuela de Agudos Dr. Ramón Madariaga. Posadas, Misiones – Argentina, posadas, Misiones, Argentina; Hospital Escuela de Agudos Dr Ramón Madariaga, Posadas, Misiones, Argentina, Posadas, Misiones, Argentina; Madariaga Hospital, Posadas, Misiones, Argentina; Infectious Diseases Department, Hospital Escuela de Agudos Dr. Ramón Madariaga. Posadas, Misiones – Argentina, posadas, Misiones, Argentina; Infectious Diseases Department, Hospital Escuela de Agudos Dr. Ramón Madariaga. Posadas, Misiones – Argentina, posadas, Misiones, Argentina; Infectious Diseases Department, Hospital Escuela de Agudos Dr. Ramón Madariaga. Posadas, Misiones – Argentina, posadas, Misiones, Argentina; Infectious Diseases Department, Hospital Escuela de Agudos Dr. Ramón Madariaga. Posadas, Misiones – Argentina, posadas, Misiones, Argentina; Infectious Diseases Department, Hospital Escuela de Agudos Dr. Ramón Madariaga. Posadas, Misiones – Argentina, posadas, Misiones, Argentina

## Abstract

**Background:**

Infective endocarditis (IE) is a serious condition with a broad range of clinical manifestations. Despite advances in diagnostic and therapeutic strategies, its morbidity and mortality have not significantly decreased over the years.

**Methods:**

We conducted a retrospective, descriptive study of cases meeting the modified Duke criteria for definite IE from 2010 to 2020. Our aim was to describe the clinical and epidemiological characteristics, complications, and mortality among this patient cohort.

**Results:**

A total of 113 patients with definite IE were included. Male sex: 64%. Median age: 48.6 years (range 17–87). Comorbidities: chronic kidney disease 15.8%, heart failure 12.3%, DM 12%. Preexisting cardiovascular conditions: rheumatic heart disease: 9.6%, valvular disease 37.5% and aortic regurgitation 55.5%. IE Localization: aortic valve: 44% (87.7% native), mitral valve: 39.8% (88.8% native), tricuspid valve: 10.6%, pulmonary valve: 0.88%, mural endocardium: 0.88%. Diagnostic modalities: TTE 79%, TEE 81.4%. Microbiological findings: positive blood cultures 86.7%. Most frequent pathogens: *Staphylococcus aureus* (33.6%), *Streptococcus spp.* (27.5%) and *Enterococcus spp.* (16.3%). In-hospital complications: persistent positive blood cultures: 28.3%, persistent fever despite appropriate antibiotic therapy: 26.5%, new neurological events: 23.8%. Surgical management: indication for surgery 57.2%, underwent surgery 75.3% (elective 36.7%, urgent: 32.6%, emergent: 30.6%). Overol mortality: 38%. Device-associated IE mortality: 25%. Factors associated with increased mortality: HF at admission (*p* = 0.004), persistent bacteremia (*p* = 0.002), worsening HF (*p* < 0.0001).

**Conclusion:**

In this 10-year retrospective study, IE affected native aortic valves, with *S. aureus* and *Streptococcus spp.* as the most common pathogens. Relatively young median age (48 y) and high overall mortality (38%) reflect the local demographic context and possible delays in diagnosis and treatment. Improving access to early diagnostic tools and specialized care is essential to reduce morbidity and mortality in this setting.

**Disclosures:**

All Authors: No reported disclosures

